# Geographic distribution of sex chromosome polymorphism in *Anastrepha fraterculus* sp. 1 from Argentina

**DOI:** 10.1186/s12863-020-00944-1

**Published:** 2020-12-18

**Authors:** María Cecilia Giardini, Mariela Nieves, Alejandra Carla Scannapieco, Claudia Alejandra Conte, Fabián Horacio Milla, María Elena Schapovaloff, Maria Soledad Frissolo, María Isabel Remis, Jorge Luis Cladera, Silvia Beatriz Lanzavecchia

**Affiliations:** 1 Laboratorio de Insectos de Importancia Agronómica, Instituto de Genética (IGEAF), Instituto de Agrobiotecnología y Biología Molecular (IABIMO), INTA- CONICET, Hurlingham, Buenos Aires, Argentina; 2 Grupo de Investigación en Biología Evolutiva, Departamento de Ecología, Genética y Evolución, IEGEBA (CONICET), Facultad de Ciencias Exactas y Naturales, Universidad de Buenos Aires, Buenos Aires, Argentina; 3 Consejo Nacional de Investigaciones Científicas y Técnicas (CONICET), Buenos Aires, Argentina; 4 Estación Experimental Agropecuaria Montecarlo, Instituto Nacional de Tecnología Agropecuaria (INTA), Misiones, Argentina; 5 Subprograma La Rioja, Programa Nacional de Control y Erradicación de Moscas de los Frutos (PROCEM), La Rioja, Argentina; 6 Genética de la Estructura Poblacional, Departamento de Ecología, Genética y Evolución,IEGEBA (CONICET), Facultad de Ciencias Exactas y Naturales, Universidad de Buenos Aires, Buenos Aires, Argentina

**Keywords:** Karyomorphs, Karyotypic polymorphism, Fruit fly pest, Dispersion patterns, Morphotypes, SIT

## Abstract

**Background:**

*Anastrepha fraterculus* is recognized as a quarantine pest in several American countries. This fruit fly species is native to the American continent and distributed throughout tropical and subtropical regions. It has been reported as a complex of cryptic species, and at least eight morphotypes have been described. Only one entity of this complex, formerly named *Anastrepha fraterculus* sp. 1, is present in Argentina. Previous cytogenetic studies on this morphotype described the presence of sex chromosome variation identified by chromosomal size and staining patterns. In this work, we expanded the cytological study of this morphotype by analyzing laboratory strains and wild populations to provide information about the frequency and geographic distribution of these sex chromosome variants. We analyzed the mitotic metaphases of individuals from four laboratory strains and five wild populations from the main fruit-producing areas of Argentina, including the northwest (Tucumán and La Rioja), northeast (Entre Ríos and Misiones), and center (Buenos Aires) of the country.

**Results:**

In wild samples, we observed a high frequency of X_1_X_1_ (0.94) and X_1_Y_5_ (0.93) karyomorphs, whereas X_1_X_2_ and X_1_Y_6_ were exclusively found at a low frequency in Buenos Aires (0.07 and 0.13, respectively), Entre Ríos (0.16 and 0.14, respectively) and Tucumán (0.03 and 0.04, respectively). X_2_X_2_ and X_2_Y_5_ karyomorphs were not found in wild populations but were detected at a low frequency in laboratory strains. In fact, karyomorph frequencies differed between wild populations and laboratory strains. No significant differences among *A. fraterculus* wild populations were evidenced in either karyotypic or chromosomal frequencies. However, a significant correlation was observed between Y_5_ chromosomal frequency and latitude.

**Conclusions:**

We discuss the importance of cytogenetics to understand the possible route of invasion and dispersion of this pest in Argentina and the evolutionary forces acting under laboratory conditions, possibly driving changes in the chromosomal frequencies. Our findings provide deep and integral genetic knowledge of this species, which has become of relevance to the characterization and selection of valuable *A. fraterculus* sp. 1 strains for mass rearing production and SIT implementation.

**Supplementary Information:**

The online version contains supplementary material available at 10.1186/s12863-020-00944-1.

## Background

The South American fruit fly, *Anastrepha fraterculus* Wiedemann (Diptera, Tephritidae), exhibits a broad geographic distribution in the American continent, ranging from 27° N to 35° S latitudes [1–5]. This pest has a wide range of host fruits, including wild and economically important plant species [5–7].

*A. fraterculus* constitutes a complex of cryptic species, with at least eight described morphotypes [8–11] and its putative center of origin is located in South America [12–14]. Integrative taxonomic studies have proposed a new perspective to study the members of *A. fraterculus* complex [15–19]. These studies have based their approaches on previous significant contributions, including the use of morphometry [9–11], cytogenetic analyses ([12, 20]; reviewed by Zacharopoulou et al. [21]), population genetics [12, 22–29], behavioral and physiological studies [30–35] and, pheromone and cuticle hydrocarbon composition analysis [36–38].

In Argentina, only one entity of this complex is present, formerly named *Anastrepha fraterculus* sp. 1 or Brazilian 1 morphotype [12, 20, 39]. This morphotype carries a karyotype composed of five pairs of acrocentric autosomes and a pair of sex chromosomes (2n = 12). Previous works performed in Argentinian wild populations described an occasional sex chromosome polymorphism ([40–42], reviewed by Cladera et al. [43]; Giardini et al. [44]). Particularly, these studies described the presence of five morphological variants of the X chromosome and four variants of the Y chromosome, with both types of polymorphism being detected at a low frequency [40–42]. Based on chromosomal size and staining patterns, later exhaustive studies have described cytotypes (or karyomorphs) composed of two variants of each sex chromosome (named X_1_, X_2_ and Y_5_, Y_6_) [45]. The X_1_ variant is a large submetacentric chromosome with two DAPI- positive bands located at each of its telomeres, the distal band being more prominent than the proximal one [20, 44–46]. The X_2_ variant is a large submetacentric chromosome with a DAPI- positive distal satellite. Its telomeric regions show the same DAPI staining patterns as the X_1_ chromosome [40, 41, 45, 47]. The Y_5_ is a small meta-submetacentric chromosome (40% shorter than X_1_) with an interstitial DAPI- positive region located in the long arm and a large DAPI- positive band in the short arm [44, 45]. The Y_6_ variant is a medium-size submetacentric chromosome 20% shorter than X_1_. This variant shows DAPI- positive bands in almost 50% of its length [45, 47]. It is worth noting that the karyomorphs identified in *A. fraterculus* sp. 1 from Argentina have shown cytological differences from those previously described for other members of the *A. fraterculus* complex [12, 20].

The existing partitioned information about the current distribution of *A. fraterculus* individuals carrying sex chromosomal variants of this morphotype, in conjunction with the uncertain taxonomic status of this species complex in America, carries important implications for the development of species- specific control strategies, such as the sterile insect technique (SIT) ([16, 17, reviewed in [13, 18]). In this context, cytogenetics plays a key role in the understanding of sex chromosome evolution and cryptic species resolution, and it is critical in the development and evaluation of SIT strategies (reviewed by Zacharopoulou et al. [21]).

In the present work, we studied the geographic distribution of sex chromosome variation in wild populations of *A. fraterculus* sp*.* 1 from Argentina and complemented this information by the analysis of laboratory strains in order to characterize chromosomal variants found at a low frequency. We discuss our results in the light of previous cytogenetic studies to understand the possible route of introduction and dispersion of this pest in Argentina. In addition, we propose some hypotheses about the possible origin of the sex chromosome variants detected so far in Argentinian populations of *A. fraterculus*. Our findings contribute to a better genetic knowledge of this species in the context of the identification of members in the *A. fraterculus* complex, thus providing tools to develop and apply environmentally safe control strategies against this fruit fly pest in Argentina and other South American countries.

## Results

**Fig. 1:**
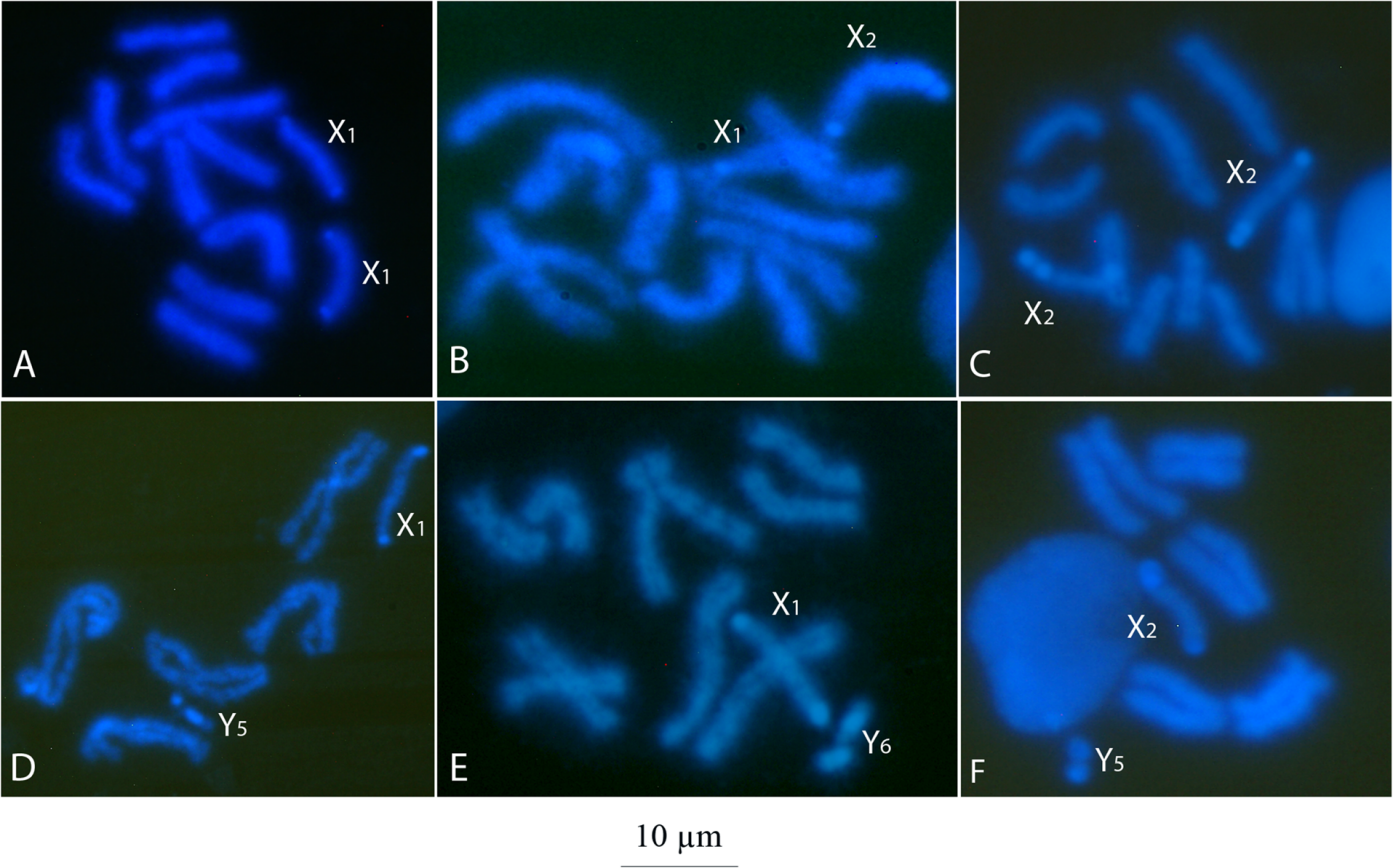
Sex chromosome karyomorphs detected in wild populations and laboratory strains of *A. fraterculus* sp. 1 from Argentina. **a-e** Cytological preparations of mitotic chromosomes stained with DAPI. **a-c** female metaphases, **d-f** male metaphases. Bar represents 10 μm

**Fig. 2:**
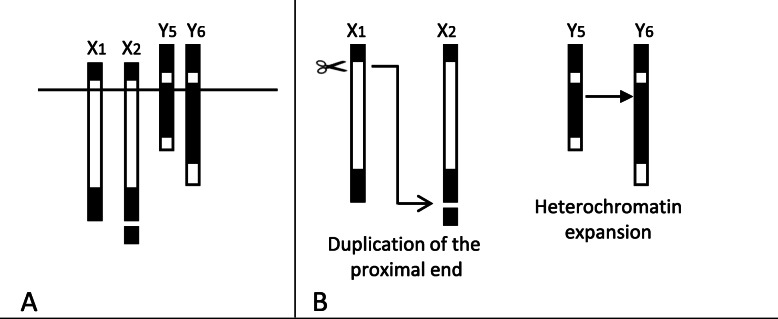
**a.** Schematic representation of sex chromosomes detected in wild and lab populations of *A. fraterculus*. Banding pattern corresponds to DAPI staining and C Bands. The line crossing all chromosome schemes shows the position of the centromere according to Giardini et al. [44]. **b.** Suggested chromosome rearrangements of X_1_ and Y_5_ to generate X_2_ and Y_6_, respectively

We analyzed 424 preparations of mitotic chromosomes of *A. fraterculus* (each made from the brain ganglia of an individual larva) and observed the presence of two size variants of X chromosome (X_1_ and X_2_; Fig. 1 a, b, and c and Fig. 2 a) and Y chromosome (Y_5_ and Y_6_; Fig. 1 d, e, and F and Fig. 2 a) in both, wild population and laboratory strain samples (Table 1; Additional File 1). In addition, no size polymorphism was detected in the autosomal complement.

**Table 1:** Relative frequency of karyomorphs detected in wild populations and laboratory strains of *A. fraterculus* sp. 1 from Argentina

Origin/Locality	Karyomorphs								
	Female				Male				
	X_**1**_X_**1**_	X_**1**_X_**2**_	X_**2**_X_**2**_	N	X_**1**_Y_**5**_	X_**1**_Y_**6**_	X_**2**_Y_**5**_	X_**2**_Y_**6**_	N
*Wild populations*									
Misiones	1.00	0.00	0.00	**17**	1.00	0.00	0.00	0.00	**13**
Tucumán	0.97	0.03	0.00	**34**	0.97	0.03	0.00	0.00	**29**
La Rioja	1.00	0.00	0.00	**4**	1.00	0.00	0.00	0.00	**6**
Entre Ríos	0.84	0.16	0.00	**19**	0.86	0.14	0.00	0.00	**22**
Buenos Aires	0.93	0.07	0.00	**14**	0.87	0.13	0.00	0.00	**15**
**Total**	**83**	**5**	**0**	**88**	**79**	**6**	**0**	**0**	**85**
*Laboratory strains*									
Af-IGEAF	0.72	0.28	0.00	**57**	0.78	0.22	0.00	0.00	**37**
Af-Y-short	0.96	0.04	0.00	**55**	1.00	0.00	0.00	0.00	**45**
Af-Cast-1	0.76	0.06	0.18	**17**	0.73	0.00	0.27	0.00	**15**
Af-Cast-2	0.93	0.00	0.07	**14**	1.00	0.00	0.00	0.00	**11**
**Total**	**120**	**19**	**4**	**143**	**96**	**8**	**4**	**0**	**108**

**Fig. 3:**
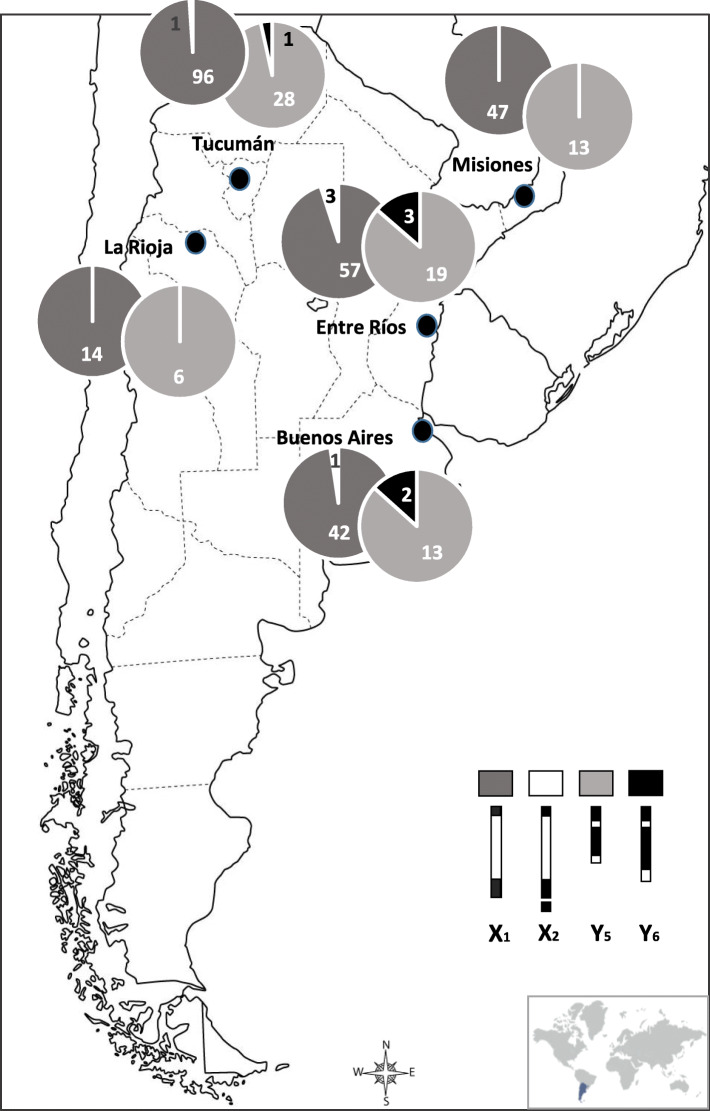
Geographic distribution and relative frequency of sex chromosome variants detected in Argentinian *A. fraterculus* wild populations (see details in Additional File 1). Numbers in or over the pie-shaped charts correspond to the absolute frequency of each chromosome variant

Specifically, for wild population samples, La Rioja and Misiones only showed one of two mitotic karyomorphs (X_1_X_1_ and X_1_Y_5_ in females and males, respectively). Samples from Buenos Aires, Tucumán, and Entre Ríos showed the presence of four different karyomorphs (X_1_X_1_/ X_1_X_2_ and X_1_Y_5_/ X_1_Y_6_, in females and males, respectively) (Table 1; Fig. 3).

The presence of X_2_Y_5_ karyomorph was only observed in the laboratory strain Af-Cast-1, while X_2_X_2_ karyomorph was detected in two laboratory strains (Af-Cast-1 and Af-Cast-2 strains of *A. fraterculus* harboring different *Wolbachia* strains). X_2_Y_6_ was not found in any of the analyzed samples (Table 1).

No significant differences were found between observed and expected karyomorph frequencies in either wild populations or laboratory strains (Fisher’s Exact Test; *p* > 0.05 in all cases). Moreover, the analysis of chromosome incidence revealed homogeneity of X variant frequencies in both sexes in nature (Fisher’s Exact Test; *p* > 0.05 in all cases). Both results mentioned above agree with Hardy Weinberg Equilibrium within each population.

The presence of X_1_X_1_ and X_1_Y_5_ karyomorphs were observed at a high frequency in all wild populations (mean frequency values: 0.94 and 0.93, respectively) (Table 1). The analysis of geographic chromosome variation revealed that there were no significant differences in either X or Y variant frequencies among wild populations (Fisher’s Exact Test; *p* > 0.05; *p* = 0.34, *p* = 0.42, respectively). Additionally, non-significant differences were found in female karyomorph frequency among wild populations (Fisher’s Exact Test; *p* = 0.2847).

The correlation analysis between chromosome frequencies from *A. fraterculus* wild populations and geographic variables (latitude and longitude) showed a significant and negative association between Y_5_ frequency and latitude (Pearson’s Correlation; *r* = 0.88; *p* = 0.0489). Conversely, Y_6_ frequency increased with the latitude (Fig. 3).

The cytogenetic characterization of laboratory strains indicated some differences with respect to wild populations. After the analysis of 94 mitotic chromosome preparations (57 females and 37 males) from the Af-IGEAF strain, significantly lower frequencies of X_1_X_1_ (0.72) and X_1_Y_5_ (0.78) and higher frequencies of X_1_X_2_ (0.28) and X_1_Y_6_ (0.22) were observed with respect to wild samples (Table 1). In fact, Fisher’s Exact Test revealed that Af-IGEAF strain exhibited significant differences in X variants (*p* = 0.0034) compared to its source wild population (Tucumán). The differences in Y variants between these samples were marginally significant (*p* = 0.06) (Additional File 1).

In the Af-Y-short strain (purified *A. fraterculus* strain harboring Y_5_ chromosome), 96% of females carried X_1_X_1_ and 4% carried X_1_X_2_ karyomorphs while 100% of males showed X_1_Y_5_, as expected for this line (Table 1). A significant increase in the frequency of the X_1_ variant was verified in Af-Y-short strain in comparison with in relation to Af-IGEAF strain (Fisher’s Exact Test; *p* = 0.0004) (Additional File 1).

Af-Cast-1 and Af-Cast-2 strains showed a differential distribution of karyomorphs (Table 1). For Af-Cast-1 strain, we detected the presence of X_1_X_1_ (76.5%), X_2_X_2_ (17.6%), and X_1_X_2_ (5.9%) in females and X_1_Y_5_ (73%) and X_2_Y_5_ (27%) in males (Table 1). For Af-Cast-2 strain, we detected the female karyomorphs X_1_X_1_ (93%) and X_2_X_2_ (7%), and no heterozygous females (X_1_X_2_) were observed. Concerning male chromosome combinations, we observed 100% of X_1_Y_5_. In addition, the mentioned strains differed significantly in their X variant frequencies (Fisher’s Exact Test; *p* = 0.0328) (Additional File 1).

## Discussion

In the present work, we studied the frequency and distribution of sex chromosome variants found in laboratory colonies and wild populations of *A. fraterculus* sp. 1 from different regions of Argentina by analyzing mitotic chromosome preparations.

The cytogenetic characterization of *A. fraterculus* sp*.* 1 wild populations located in different eco-climatic regions representing the main fruit-producing areas of Argentina allowed us to identify four sex chromosome cytotypes (or karyomorphs) (X_1_X_1_/ X_1_Y_5_/ X_1_X_2_/ X_1_Y_6_) and the absence of individuals harboring X_2_X_2_, X_2_Y_5,_ and X_2_Y_6_ karyomorphs. These techniques were not useful in detecting chromosomal variation in the autosomes of the analyzed populations. Our results were slightly different from those previously reported by Lifschitz et al. [40], Manso and Basso [41], Basso et al. [42], and more recently by Basso et al. [48, 49]. These studies described the presence of several variants of X (X_1_, X_2_, X_3_, X_4_) and Y (Y_1_, Y_2_, Y_3_, Y_4_, Y_5_, Y_6_) chromosomes in *A. fraterculus* from Argentina. However, this variation was not observed in the extensive sampling of wild populations performed for the present work.

Concerning the karyomorph characterization of established laboratory colonies, we observed that Af-IGEAF laboratory strain showed significant differences in the distribution of chromosomal combinations compared to the current frequency of its founding wild population (Tucumán). This could be the consequence of stochastic and/or artificial selection effects driving changes in the chromosome and karyotypic frequencies. Similar processes were previously described for this species during the laboratory adaptation [50] and also observed in other Tephritidae species [51, 52]. Indeed, the other three laboratory strains analyzed here showed biased frequencies of chromosomal variants, as expected for these types of laboratory colonies, founded from Af-IGEAF strain with specific purposes and, using less than 50 parental crosses. In Af-Cast-1 and Af-Cast-2 strains (*A. fraterculus* colonies harboring different *Wolbachia* strains), we observed the presence of karyomorphs absent in wild populations (X_2_X_2_/ X_2_Y_5_). It is worth noting that X_2_Y_6_ was not observed in any of the colonies or wild populations analyzed, mainly explained by the low chromosomal frequency of Y_6_ detected in them. However, these less frequent or absent karyomorphs in adult individuals and possible chromosome incompatibilities associated to the presence of *Wolbachia* need further analyses of paired-crosses, including parameters such as fecundity and larval survival as were previously evaluated in other insect species [53–56].

The analysis of both chromosome and karyomorph frequencies registered for wild *A. fraterculus* populations showed no differences among the studied localities but evidenced a significant trend of a differential distribution of the chromosome frequencies. In particular, a negative correlation was observed for the Y_5_ distribution according to latitude. The information available with respect to the distribution of *A. fraterculus* morphotypes in South America and the cytological studies previously performed, in conjunction with the results described here, can be of help to put forward some hypotheses about the introduction and dispersion of *A. fraterculus* sp*.* 1 in the Argentine territory. Recent studies proposed a possible non-monophyletic origin of *A. fraterculus* in South America. The expansion of this species to different regions of the South American subcontinent may have initiated by two unconnected routes of invasion: One arm extended along the western edge, including both highland and lowland areas of the Andean region, and the other along the eastern Brazilian coast [12–14]. In this sense, we consider that *A. fraterculus* Brazilian 1 morphotype could have entered Argentina through the northeast (Misiones) from Brazil. This movement is expected for this *A. fraterculus* morphotype, due to the geographic proximity, and it is evidenced by a conserved karyomorph (previously described by Selivon et al. [12] and Goday et al. [20] for *A. fraterculus* from Brazil and by Manso and Basso [41] for *A. fraterculus* from Argentina). Another probable route of invasion is through the northwest of the country (Jujuy-Tucumán) by the Peruvian *A. fraterculus*. The Peruvian karyotype was first described by Cáceres et al. [15] and is similar to that previously described for the Ecuatorian morphotype [20]. The cytological analysis of the Peruvian morphotype showed sex chromosomes of similar length, designated X_p_ and Y_p_. The X_p_ chromosome has a prominent interstitial heterochromatic block, whereas the Y_p_ chromosome has a DAPI- positive block located at the centromeric region of the chromosome [15].

In our analysis of 173 *A. fraterculus* individuals belonging to Argentinian wild populations, we did not observe karyomorphs similar to those described for the Peruvian morphotype. Furthermore, the currently available information does not provide enough cytogenetic evidence to describe possible hybridization events between Brazilian 1 and Peruvian morphotypes, like those previously described by Selivon et al. [12, 57] and Cáceres et al. [15] through laboratory-controlled crosses. Although the results shown here support the assumption of a unique origin of this *A. fraterculus* sp. 1 in Argentina, further cytogenetic analysis (including populations from Brazil and western South American countries) in conjunction with genetic and morphological studies could contribute to our knowledge about possible routes of invasion of this pest in Argentina.

Another key point we address here is the potential source of the sex chromosome polymorphism detected in *A. fraterculus* from Argentina. We propose an explanation for the generation of less frequent X_2_ and Y_6_ variants as possibly caused by modifications in X_1_ and Y_5_ chromosomes, respectively. These sex chromosome variants were previously described as forming the unique karyomorph of *A. fraterculus* sp. 1 (X_1_Y_5_) [12, 20]. The X_2_ chromosome could be derived from X_1_ by a duplication of the proximal heterochromatic block followed by a chromosome breakage and a subsequent cohesion to the distal telomeric region, giving rise to the X_2_ heterochromatic satellite (Fig. 2b). This hypothesis is supported by previous studies on chromosome behavior during cell division [58, 59]. Throughout this cell process, centromeres adopt a complex structure that makes them susceptible to be the site of chromosome rearrangements, as reviewed by Barra and Fachinetti [60]. These authors support the hypothesis that the most probable chromosome site to suffer duplication and/or breakage to form the X_2_ satellite is the proximal and pericentromeric zone of the X_1_ chromosome. On the other hand, the Y_6_ variant could be derived from Y_5_ by duplication and expansion of the larger heterochromatic block (Fig. 2b). Previous studies described the behavior of constitutive heterochromatin as dynamically regulated [61]. In addition, transitions between both types of chromatin (euchromatin and heterochromatin) were previously described for telomeric heterochromatin and satellite DNA in *Drosophila* [62], supporting our hypothesis of interstitial heterochromatin expansion to form the Y_6_ variant.

No further information regarding this type of intra-morphotype variation has been reported in other members of this species complex so far. Future studies using integrated standard cytogenetic techniques, FISH (fluorescence in situ hybridization), CGH (comparative genomic hybridization), mapping of major ribosomal RNAs (rRNAs), and H3 histone genes will contribute to understand the nature of this variation and the chromosomal evolution of this morphotype. These techniques could also be useful to analyze the role of the detected polymorphism on the speciation process of *A. fraterculus* and the dispersion patterns of cryptic species in America.

Cytogenetics has played an essential role in integrative taxonomic studies that clarify relationships between closely related species and/or incipient speciation phenomena [21, 63, 64] and has been used in the development and application of SIT for major Tephritidae species [reviewed in 21]. In particular, the knowledge of mitotic and polytene chromosomes has been applied to the construction and characterization of classical genetic sexing strains [65–67]. In addition, the chromosome characterization has significantly contributed to recent genome projects of tephritid pest species and made it possible to identify the linkage groups facilitating genome assemblies [68, 69].

## Conclusions

This study provides relevant information about the sex chromosome polymorphism in *A. fraterculus* sp. 1 from Argentina and describes possible routes of invasion and dispersion of this pest species in the territory. Although previous studies have not reported intra-morphotype variation at the chromosomal level in other members of the *A. fraterculus* complex so far, we consider that a deeper cytogenetic analysis of these wild populations, including mitotic and polytene chromosomes analyses, will greatly contribute to shedding light on the origin and evolution of this complex. Moreover, the establishment of standardized protocols of integrative taxonomy for this cryptic species complex may allow the univocal identification of species and, therefore, the development of specific control strategies at the regional level. Detailed activities performed following the same guidelines in different laboratories of South America, organized in a common database and including multidisciplinary studies (e.g., morphometry, cytogenetics, phylogenetic, ecological and behavioral parameters, eco-chemistry, and genetics), in conjunction with the study of reproductive symbionts, seem to be the best strategy to address the complexity of the *A. fraterculus* complex.

## Methods

### Insects

Wild *A. fraterculus* individuals (larvae) were obtained from infested fruit species available in each sampling site, distributed in different eco-climatic regions and representing the fruit-producing area of Argentina (Table 1; Fig. 3). The fruit was collected during three consecutive fruiting seasons (2016–2018). The sampling sites, ordered by geographic coordinates were as follows: Montecarlo, Misiones ([26°33′58.32“ S 54°45’25.2” W]; fruit species sampled: guava [*Psidium guajava*]); Horco Molle, Tucumán ([26°49′0″ S 65°19′0″ W]; fruit species sampled: peach [*Prunus persica*] and guava); San Blas de los Sauces, La Rioja ([28°24′37.84“ S 67°5’36.28” W]; fruit species sampled: peach and plum [*Prunus domestica*]); Concordia, Entre Ríos ([31°23′34.66“ S 58°1’15.2” W]; fruit species sampled: peach and guava); Hurlingham, Buenos Aires ([34°35′17.92“ S 58°38’20.58” W]; fruit species sampled: peach and plum).

The infested fruits were kept at a quarantine room with controlled conditions of temperature and relative humidity (25 ± 1 °C and 70 ± 10%) until *A. fraterculus* 3rd-instar larvae were recovered. The species identification was based on morphological characteristics (shape and number of tubules) of anterior spiracles, according to Frias et al. [70].

### Laboratory strains

Immature stages of *A. fraterculus* from the following laboratory strains were included in the cytological analysis.

#### Af-IGEAF strain

This colony (named afterward Af IGEAF) was established in 2007 with approximately 10,000 pupae from the semi-mass rearing colony kept at Estación Experimental Agroindustrial Obispo Colombres, San Miguel de Tucumán, Tucumán, Argentina [71] and maintained to date (120 generations) under artificial rearing.

#### Af-Y-short strain

This strain was purified from the Af IGEAF strain and it harbors Y_5_ chromosome (the shortest Y chromosome reported for this species). This colony was founded after the screening of 25 families, originally composed of one parental male and three females. After analyzing all the families, we pooled those with the Y_5_ chromosome. This strain was maintained for 70 generations under laboratory conditions.

#### Af-Cast-1 and Af-Cast-2 strains

These two *A. fraterculus* lines were also purified from the *A. fraterculus* IGEAF strain, considering the *Wolbachia* strain they harbor (*w*AfraCast1_A and *w*AfraCast2_A, respectively) [72]. Each strain was maintained for 70 generations under laboratory conditions.

##### Preparations and staining of mitotic chromosomes

We followed the cytological technique described by Guest and Hsu [73] with minor modifications. Briefly, cerebral ganglia of *A. fraterculus* 3rd-instar larvae were dissected in Ringer solution and incubated in hypotonic solution (1% sodium citrate) for 10–15 min. The material was fixed for 1 min in freshly prepared fixative (methanol-acetic acid, 3:1) and then homogenized in 60% (v/v) acetic acid with a micropipette. For each preparation, the homogenized suspension was dropped onto a clean slide, which was placed on a hot plate to allow the tissue to spread, and then, air-dried. After drying, the preparations were immersed in DAPI solution (50 ng/ml in 2x SSC) for 5–7 min. Slides were mounted in antifade and observed under an Olympus BX40 (Olympus, Tokyo, Japan) microscope at 1000X magnification.

##### Data analysis

Analyses of chromosome and karyomorph frequencies among wild populations or laboratory strains were performed using Fisher’s Exact Test. Hardy Weinberg Equilibrium (HWE) for X chromosome variants, is characterized by both homogeneity of variant frequencies between sexes and Hardy Weinberg proportions in females [74]. We verified HWE deviations through Fisher’s Exact Tests by comparing both i) X chromosome variant frequencies between males and females and ii) observed and excepted karyomorph frequencies in females. Fisher’s Exact Tests with *p*-value computed based on the network developed by Mehta and Patel [75] were implemented in the R package [76]. The relationship between chromosome variant frequencies and geographic variables (latitude and longitude) in wild populations was assessed through the analysis of Pearson’s correlation coefficient in Infostat Professional version 2014 [77].

## Supplementary Information

**Additional file 1. **Relative frequency of sex chromosome variants detected in wild and laboratory strains of *A. fraterculus* sp. 1 from Argentina.

## Data Availability

The wild material described in this work was obtained from infested fruit collections as it was mentioned in the Methods section. The laboratory lines studied were from the Laboratorio de Insectos de Importancia Agronómica, Instituto de Genética (INTA) Buenos Aires, Argentina.

## Abbreviations

CGH: Comparative genomic hybridization
DAPI: 4′ 6-diamidino-2-phenylindole
FISH: Fluorescence in situ hybridization
HWE: Hardy-Weinberg equilibrium
min: Minutes
N: North
rRNA: Ribosomal RNA
S: South
SIT: Sterile Insect Technique
sp.: Specie
W: Western

## References

[1] Stone A. The fruit flies of the genus Anastrepha. USDA Misc Publ. Washington DC, USA, Publication 439; 1942.

[2] Hernández-Ortiz V, Aluja M (1993). Listado de especies del género neotropical Anastrepha (Diptera: Tephritidae) con notas sobre su distribución y plantas hospederas. Folia Entomol Mex.

[3] Norrbom AL, Zucchi RA, Hernández-Ortiz V, Aluja M, Norrbom AL (1999). Phylogeny of the genera Anastrepha and Toxotrypana (Trypetinae: Toxotrypanini) based on morphology. Fruit flies (Tephritidae): Phylogeny and evolution of behavior.

[4] Steck GJ (1999). Taxonomic status of *Anastrepha fraterculus*. The south American fruit fly, *Anastrepha fraterculus* (Wied.): advances in artificial rearing, taxonomic status and biological studies. IAEA-TECDOC- 1064 Vienna.

[5] Zucchi RA. Diversidad, distribución y hospederos del género Anastrepha en Brasil. In: Hérnandez-Ortiz V (ed.). Moscas de la fruta en Latinoámerica (Diptera: Tephritidae): Diversidad, biologia y manejo. S y G editores. Distrito Federal; México; 2007. p. 77–100.

[6] Zucchi RA, Moraes RCB. Fruit flies in Brazil– Anastrepha species and their host plants and parasitoids. Available in: www.lea.esalq.usp.br/anastrepha/. Accessed: 28 Nov 2019.

[7] Norrbom AL. Host plant database for Anastrepha and Toxotrypana (Diptera: Tephritidae: Toxotrypani), Diptera Data Dissemination Disk. CD 2004, − not a journal.

[8] Steck GJ (1991). Biochemical systematics and population genetic structure of Anastrepha fraterculus and related species (Diptera: Tephritidae). Ann Entomol Soc Am.

[9] Hernández-Ortiz V, Gomez-Anaya JA, Sanchez A, Mc Pheron BA, Aluja M (2004). Morphometric analysis of Mexican and South American populations of the *Anastrepha fraterculus* complex (Diptera: Tephritidae) and recognition of a distinct Mexican morphotype. Bull Entomol Res.

[10] Hernández-Ortiz V, Bartolucci AF, Morales-Valles P, Frías D, Selivon D (2012). Cryptic Species of the *Anastrepha fraterculus* Complex (Diptera: Tephritidae): A multivariate approach for the recognition of South American morphotypes. Ann Entomol Soc Am.

[11] Hernández-Ortiz V, Canal NA, Tigrero Salas JO, Ruíz-Hurtado FM, Dzul-Cauich JF (2015). Taxonomy and phenotypic relationships of the *Anastrepha fraterculus* complex in the Mesoamerican and Pacific Neotropical dominions (Diptera, Tephritidae). Zookeys.

[12] Selivon D, Perondini ALP, Morgante JS (2005). A genetic morphological characterization of two cryptic species of the Anastrepha fraterculus complex. (Diptera: Tephritidae). Ann Entomol Soc Am.

[13] Hendrichs J, Vera MT, De Meyer M, Clarke AR. Resolving cryptic species complexes of major tephritid pests. In: De Meyer M, Clarke AR, Vera MT, Hendrichs J, editors. Resolution of cryptic species complexes of Tephritid pests to enhance SIT application and facilitate international trade. ZooKeys. 2015;540:5–39. 10.3897/zookeys.540.9656. 10.3897/zookeys.540.9656 PMC4714062 26798252

[14] Mengual X, Kerr P, Norrbom AL, Barr NB, Lewis ML, Stapelfeldt AM, Scheffer SJ, Woods P, Islam MS, Korytkowski CA, Uramoto K, Rodriguez EJ, Sutton BD, Nolazco N, Steck GJ, Gaimari S (2017). Phylogenetic relationships of the tribe Toxotrypanini (Diptera: Tephritidae) based on molecular characters. Mol Phylogenet Evol.

[15] Cáceres C, Segura D, Vera MT, Wornoaypor V, Cladera JL, Teal P, Sapountzis P, Bourtzis K, Zacharopoulou A, Robinson A (2009). Incipient speciation revealed in Anastrepha fraterculus (Diptera; Tephritidae) by studies on mating compatibility, sex pheromones, hybridization, and cytology. Biol J Linn Soc.

[16] Vaníčková L, Hernández-Ortiz V, Joachim Bravo IS, Dias V, Passos Roriz AK, Laumann RA, de Lima Mendonça A, Aguiar Jordão Paranhos B, Rufino do Nascimento R (2015). Current knowledge of the species complex Anastrepha fraterculus (Diptera, Tephritidae) in Brazil. Zookeys..

[17] Dias VS, Silva JG, Lima KM, Petitinga CSCD, Hernández-Ortiz V, Laumann RA, Paranhos BJ, Uramoto K, Zucchi RA, Joaquim-Bravo IS (2016). An integrative multidisciplinary approach to understanding cryptic divergence in Brazilian species of the Anastrepha fraterculus complex (Diptera: Tephritidae). Biol J Linn Soc.

[18] Schutze MK, Virgilio M, Norrbom A, Clarke AR (2017). Tephritid integrative taxonomy: where we are now, with a focus on the resolution of three tropical fruit fly species complexes. Annu Rev Entomol.

[19] Prezotto LF, Perondini ALP, Hernández-Ortiz V, Frías D, Selivon D (2019). What can integrated analysis of morphological and genetic data still reveal about the *Anastrepha fraterculus* (Diptera: Tephritidae) cryptic species complex?. Insects.

[20] Goday C, Selivon D, Perondini ALP, Graciano PG, Ruiz MF (2006). Cytological characterization of sex chromosomes and ribosomal DNA location in Anastrepha species (Diptera: Tephritidae). Cytogenet Genome Res.

[21] Zacharopoulou A, Augustinos AA, Drosopoulou E, Tsoumani KT, Gariou-Papalexiou A, Franz G, Mathiopoulos KD, Bourtzis K, Mavragani-Tsipidou P (2017). A review of more than 30 years of cytogenetic studies of Tephritidae in support of sterile insect technique and global trade. Entomol Exp Appl.

[22] Morgante JS, Malavasi A, Bush GL (1980). Biochemical systematics and evolutionary relationships of neotropical Anastrepha. Ann Entomol Soc Am.

[23] Steck GJ, Sheppard WS, Aluja M, Liedo P (1993). Mitochondrial DNA variation in Anastrepha fraterculus. Fruit Flies.

[24] Alberti AC, Calcagno G, Saidman BO, Vilardi JC (1999). Analysis of the genetic structure of a natural population of *Anastrepha fraterculus* (Diptera: Tephritidae). Ann Entomol Soc Am.

[25] MRB S-C, BA MP, Silva JG, Zucchi RA (2001). Phylogenetic relationships among species of the fraterculus group (Anastrepha: Diptera: Tephritidae) inferred from DNA sequences of mitochondrial cytochrome oxidase 1. Neotrop Entomol.

[26] Selivon D, Perondini ALP. Especies crípticas del complejo Anastrepha fraterculus en Brasil. In: Hernández-Ortiz V (ed.). Moscas de la fruta en Latinoamérica (Diptera: Tephritidae): diversidad, biológia y manejo. S y G editores, Distrito Federal, México. 2007;101–118.

[27] Ludeña CE. Agricultural productivity growth, Efficiency Change and Technical Progress in Latin America and the Caribbean. Inter-American Development Bank. 2010. www.iadb.org.

[28] Sutton BD, Steck GJ, Norrbom AL, Rodriguez EJ, Srivastava P, Nolazco Alvarado N, Colque F, Yábar Landa E, Lagrava Sánchez JJ, Quisberth E, Arévalo Peñaranda E, Rodriguez Clavijo PA, Alvarez-Baca JK, Guevara Zapata T, Ponce P (2015). Nuclear ribosomal internal transcribed spacer 1 (ITS1) variation in the Anastrepha fraterculus cryptic species complex (Diptera, Tephritidae) of the Andean region. ZooKeys..

[29] Barr NB, Ruiz-Arce R, Farris RE, Gomes Silva J, Lima KM, Siqueira Dutra V, Ronchi-Teles B, Kerr PH, Norrbom AL, Nolazco N, Thomas DB (2017). Identifying Anastrepha (Diptera; Tephritidae) species using DNA barcodes. J Econ Entomol.

[30] Vera MT, Cáceres C, Wornoayporn V, Islam A, Robinson AS, De La Vega MH, Hendrichs J, Cayol JP (2006). Mating incompatibility among populations of the south American fruit fly Anastrepha fraterculus (Diptera: Tephritidae). Ann Entomol Soc Am.

[31] Segura D, Vera MT, Rull J, Wornoayporn V, Ismal I, Robinson AS (2011). Assortative mating among Anastrepha fraterculus (Diptera: Tephritidae) hybrids as a possible route to radiation of the fraterculus cryptic species complex. Biol J Linn Soc.

[32] Rull J, Abraham S, Kovaleski A, Segura DF, Islam A, Wornoayporn V, Dammalage T, Santo Tomas U, Vera MT (2012). Random mating and reproductive compatibility among Argentinean and southern Brazilian populations of Anastrepha fraterculus (Diptera: Tephritidae). B Entomol Res.

[33] Rull J, Abraham S, Kovaleski A, Segura DF, Mendoza M, Liendo C, Vera MT (2013). Evolution of prezygotic and post- zygotic barriers to gene flow among three cryptic species within the Anastrepha fraterculus complex. Entomol Exp Appl..

[34] Devescovi F, Abraham S, Passos Roriz AK, Nolazco N, Castañeda R, Tadeo E, Cáceres C, Segura DF, Vera MT, Joachim-Bravo I, Canal N, Rull J (2014). Ongoing speciation within the Anastrepha fraterculus cryptic species complex: the case of the Andean morphotype. Entomol Exp Appl..

[35] Dias VS, Hallman GJ, AAS C, Hurtado NV, Rivera C, Maxwell F, Cáceres-Barrios CE, MJB V, Myers SW. Relative tolerance of three morphotypes of the *Anastrepha fraterculus* complex (Diptera: Tephritidae) to cold phytosanitary treatment. J Econ Entomol. 2020. 10.1093/jee/toaa027. 10.1093/jee/toaa027 PMC7275689 32161970

[36] Břízová R, Vaníčková L, Faťarová M, Ekesi S, Hoskovec M, Kalinová B (2015). Analyses of volatiles produced by the African fruit fly species complex (Diptera, Tephritidae). Zookeys..

[37] Roriz AKP, Japyassú HF, Cáceres C, Vera MT, Joachim Bravo IS (2019). Pheromone emission patterns and courtship sequences across distinct populations within Anastrepha fraterculus (Diptera-Tephritidae) cryptic species complex. Bull Entomol Res.

[38] Vaníčková L, Břízová R, Mendonça AL, Pompeiano A, Do Nascimento RR (2015). Intraspecific variation of cuticular hydrocarbon profiles in the Anastrepha fraterculus (Diptera: Tephritidae) species complex J. Appl Entomol.

[39] Zucchi RA. Taxonomia. In: Malavasi A, Zucchi RA, editors. Moscas-das-frutas de importância econômica no Brasil. Conhecimento básico e aplicado. Riberão Preto: Holos; 2000. p. 13–24.

[40] Lifschitz E, Manso FC, Basso A. Karyotype study of the south American fruit Fly, *Anastrepha fraterculus* (Wied.) in Argentina. In: American Fruit Fly, Anastrepha fraterculus (Wied.). Advances in Artificial Rearing, Taxonomic status and Biological Studies. IAEA-TECDOC-1064. ISSN 1011-4289. Austria, Vienna, 1999.

[41] Manso F, Basso A. Notes on the present situation of *Anastrepha fraterculus* in Argentina. In: IAEA, editors. The South American Fruit Fly, *Anastrepha fraterculus* (Wied.). Advances in Artificial Rearing Taxonomic Status and Biological Studies. Austria: Vienna, 1999. p. 147–162.

[42] Basso A, Sonvico A, Quesada-Allue LA, Manso F (2003). Karyotypic and molecular identification of laboratory stocks of the south American fruit fly Anastrepha fraterculus (Wied.) (Diptera: Tephritidae). J Econ Entomol.

[43] Cladera JL, Vilardi JC, Juri M, Paulin LE, Giardini MC, Gómez Cendra PV, Segura DF, Lanzavecchia SB (2014). Genetics and biology of Anastrepha fraterculus: research supporting the use of the sterile insect technique (SIT) to control this pest in Argentina. BMC Genet.

[44] Giardini MC, Milla FH, Lanzavecchia SB, Nieves M, Cladera JL (2015). Sex chromosomes in mitotic and polytene tissues of Anastrepha fraterculus (Diptera, Tephritidae) from Argentina: a review. Zookeys..

[45] Giardini MC. *Anastrepha fraterculus*: Estudios citológicos de reordenamientos cromosómicos espontáneos e inducidos en una colonia de laboratorio. Bachelor Thesis. Universidad de Buenos Aires, Buenos Aires, Argentina; 2006.

[46] Basso A, Manso FC (1998). Are Anastrepha fraterculus chromosomal polymorphisms an isolation barrier?. Cytobios..

[47] Basso A. Caracterización genética de los componentes del “complejo *Anastrepha fraterculus*” (Anastrepha spp. Diptera: Tephritinae, Trypetinae) (Wiedemann) mediante análisis de la variabilidad cromosómica. PhD Thesis, Universidad de Buenos Aires, Buenos Aires, Argentina; 2003.

[48] Basso A. Reference karyotypes and chromosomal variability: A journey with fruit flies and the key to survival. In: Chromosomal abnormalities. A hallmark manifestation of genomic instability. 2017. Chapter 9. p. 161–179.

[49] Basso A, Pereyra A, Bartolini N (2019). Chromosome- site interaction in the south American fruit fly Anastrepha fraterculus (Wied.). J Appl Biotechnol Bioeng.

[50] Parreño A, Scannapieco AC, Remis MI, Juri M, Vera MT, Segura DF, Cladera JL, Lanzavecchia SB (2014). Dynamics of genetic variability in Anastrepha fraterculus (Diptera: Tephritidae) during adaptation to laboratory rearing conditions. BMC Genet.

[51] Gilchrist AS, Cameron EC, Sved JA, Meats AW (2012). Genetic consequences of domestication and mass rearing of pest fruit fly Bactrocera tryoni (Diptera: Tephritidae). J Econ Entomol.

[52] Zygouridis NE, Argov Y, Nemny-Lavy E, Augustinos AA, Nestel D, Mathiopoulos KD (2014). Genetic changes during laboratory domestication of an olive fly SIT strain. J Appl Entomol.

[53] Lassy C, Karr T (1996). Cytological analysis of fertilization and early embryonic development in incompatible crosses of Drosophila simulans. Mech Dev.

[54] Tram U, Sullivan W (2002). Role of delayed nuclear envelope breakdown and mitosis in Wolbachia- induced cytoplasmicincompability. Science..

[55] Landmann F, Orsi GA, Loppin B, Sullivan W (2009). Wolbachia- mediated cytoplasmic incompatibility is associated with impaired histone deposition in the male pronucleus. PLoS Pathog.

[56] Beckmann J, Ronau J, Hochstrasser M (2017). A Wolbachia deubiquitylating enzyme induces cytoplasmic incompatibility. Nat Microbiol.

[57] Selivon D, Perondini ALP, Morgante JS. Haldane’s rule and other aspects of reproductive isolation observed in the *Anastrepha fraterculus* complex (Diptera: Tephritidae). Genet Mol Biol. 1999;22(4):507–10.

[58] Santaguida S, Musacchio A (2009). The life and miracles of kinetochores. EMBO J.

[59] Tanaka TU, Clayton L, Natsume T (2013). Three wise centromere functions: see no error, hear no break, speak no delay. EMBO Rep.

[60] Barra V, Fachinetti D (2018). The dark side of centromeres: types, causes and consequences of structural abnormalities implicating centromeric DNA. Nat Commun.

[61] Wang J, Jia ST, Jia S (2016). New insights into the regulation of heterochromatin. Trends Genet.

[62] Koryakov DE, Alekseyenko AA, Zhimulev IF (1999). Dynamic organization of the beta-heterochromatin in the Drosophila melanogaster polytene X chromosome. Mol Gen Genet.

[63] Drosopoulou E, Christina Pantelidou C, Gariou-Papalexiou A, Augustinos AA, Chartomatsidou T, Kyritsis GA, Bourtzis K, Mavragani-Tsipidou P, Zacharopoulou A (2017). The chromosomes and the mitogenome of Ceratitis fasciventris (Diptera: Tephritidae): two genetic approaches towards the Ceratitis FAR species complex resolution. Sci Rep.

[64] Potter S, Bragg JG, Blom MPK, Deakin JE, Kirkpatrick M, Eldridge MDB, Moritz C (2017). Chromosomal speciation in the genomics era: disentangling phylogenetic evolution of rock-wallabies. Front Genet.

[65] Zacharopoulou A (1987). Cytogenetic analysis of mitotic and salivary gland chromosomes in the medfly Ceratitis capitata. Genome..

[66] Robinson AS (2002). Mutations and their use in insect control. Mutat Res.

[67] Garcia-Martinez V, Hernandez-Ortiz E, Zepeta-Cisneros CS, Robinson AS, Zacharopoulou A, Franz G (2009). Mitotic and polytene chromosome analysis in the Mexican fruit fly, *Anastrepha ludens* (Loew) (Diptera: Tephritidae). Genome.

[68] Gilchrist AS, Shearman DCA, Frommer M, Raphael KA, Deshpande NP, Wilkins MR, Sherwin WB, Sved JA (2014). The draft genome of the pest tephritid fruit fly Bactrocera tryoni: resources for the genomic analysis of hybridising species. BMC Genomics.

[69] Papanicolaou A, Schetelig MF, Arensburger P, Atkinson PW, Benoit JB (2016). The whole genome sequence of the Mediterranean fruit fly, Ceratitis capitata (Wiedemann), reveals insights into the biology and adaptive evolution of a highly invasive pest species. Genome Biol.

[70] Frías D, Hernández-Ortiz V, Vaccaro NC, Bartolucci AF, Salles LA. Comparative morphology of immature stages of some frugivorous species of fruit flies (Diptera: Tephritidae). Isr J Entomol. 2006; 35/36:423–457.

[71] Jaldo HE, Gramajo MC, Willink E (2001). Mass rearing of Anastrepha fraterculus (Diptera: Tephritidae): a preliminary strategy. Fla Entomol.

[72] Conte CA, Segura DF, Milla FH, Augustinos AA, Cladera JL, Bourtzis K, Lanzavecchia SL (2019). Wolbachia infection in Argentinean populations of Anastrepha fraterculus sp1: preliminary evidence of sex ratio distortion by one of two strains. BMC Microbiol.

[73] Guest WC, Hsu TC (1973). A new technique for preparing Drosophila neuroblast chromosomes. Drosop Inf Serv.

[74] Hartl DL, Clark AG. Principles of population genetics. 4th ed. Sunderland, Massachusetts: Sinauer Associates; 2007.

[75] Mehta CR, Patel NR (1983). A network algorithm for performing fisher's exact test in r × c contingency tables. J Am Stat Assoc.

[76] R Core Team. R: A language and environment for statistical computing. R Foundation for Statistical Computing, Vienna, Austria. https://www.R-project.org/ (2017).

[77] Di Rienzo JA, Casanoves F, Balzarini MG et al. InfoStat versión 2014. InfoStat Group, Facultad de Ciencias Agropecuarias, Universidad Nacional de Córdoba, Argentina. http://www.infostat.com.ar (2014).

