# Supplementary material for: Geographic distribution of sex chromosome polymorphism in Anastrepha fraterculus sp. 1 from Argentina
Source: BMC Genet. 2020 Dec 18;21(Suppl 2):149. doi: 10.1186/s12863-020-00944-1 (PMC7747450; doi:10.1186/s12863-020-00944-1)
Supplement: Supplementary file 1 — Additional file 1. Relative frequency of sex chromosome variants detected in wild and laboratory strains of A. fraterculus sp. 1 from Argentina. [file 12863_2020_944_MOESM1_ESM.docx]

**Additional File 1.** Relative frequency of sex chromosome variants detected in wild and laboratory strains of *A. fraterculus* sp. 1 from Argentina.

| **Description/ Sex chromosome variants** | | **X_1_** | **X_2_** | **Nx** | **Y_5_** | **Y_6_** | **Ny** |
| --- | --- | --- | --- | --- | --- | --- | --- |
| Wild populations | Misiones | 1.00 | 0.00 | **47** | 1.00 | 0.00 | **13** |
|  | Tucumán | 0.99 | 0.01 | **97** | 0.97 | 0.03 | **29** |
|  | La Rioja | 1.00 | 0.00 | **14** | 1.00 | 0.00 | **6** |
|  | Entre Ríos | 0.95 | 0.05 | **60** | 0.86 | 0.14 | **22** |
|  | Buenos Aires | 0.98 | 0.02 | **43** | 0.87 | 0.13 | **15** |
|  |  |  |  |  |  |  |  |
| Laboratory strains | Af-IGEAF | 0.89 | 0.11 | **151** | 0.78 | 0.22 | **37** |
|  | Af-Y-short | 0.99 | 0.01 | **155** | 1.00 | 0.00 | **45** |
|  | Af-Cast-1 | 0.78 | 0.22 | **49** | 1.00 | 0.00 | **15** |
|  | Af-Cast-2 | 0.95 | 0.05 | **39** | 1.00 | 0.00 | **11** |
